# Influence of Dose on Particle Size and Optical Properties of Colloidal Platinum Nanoparticles

**DOI:** 10.3390/ijms131114723

**Published:** 2012-11-12

**Authors:** Elham Gharibshahi, Elias Saion

**Affiliations:** Department of Physics, University Putra Malaysia, 43400 UPM Serdang, Selangor, Malaysia; E-Mail: elhamgs2002@yahoo.com

**Keywords:** nanoscience, metal nanoparticle theory, conduction electrons, conduction bands, platinum nanoparticles, radiolytic method, quantum mechanical calculation

## Abstract

Attempts to produce colloidal platinum nanoparticles by using steady absorption spectra with various chemical-based reduction methods often resulted in the fast disappearance of the absorption maxima leaving reduced platinum nanoparticles with little information on their optical properties. We synthesized colloidal platinum nanoparticles in an aqueous solution of polyvinyl pyrrolidone by gamma radiolytic reduction method, which produced steady absorption spectra of fully reduced and highly pure platinum nanoparticles free from by-product impurities or reducing agent contamination. The average particle size was found to be in the range of 3.4–5.3 nm and decreased with increasing dose due to the domination of nucleation over ion association in the formation of metal nanoparticles by the gamma radiolytic reduction method. The platinum nanoparticles exhibit optical absorption spectra with two absorption peaks centered at about 216 and 264 nm and the peaks blue shifted to lower wavelengths with decreasing particle size. The absorption spectra of platinum nanoparticles were also calculated using quantum mechanical treatment and coincidently a good agreement was obtained between the calculated and measured absorption peaks at various particle sizes. This indicates that the 216 and 264-nm absorption peaks of platinum nanoparticles conceivably originated from the intra-band transitions of conduction electrons of (*n* = 5, *l* = 2) and (*n* = 6, *l* = 0) energy states respectively to higher energy states. The absorption energies, *i.e.*, conduction band energies of platinum nanoparticles derived from the absorption peaks increased with increasing dose and decreased with increasing particle size.

## 1. Introduction

The last two decades have seen remarkable progress in nanoscience and nanotechnology, particularly in the synthesis of metallic nanomaterials, aiming at better materials that have the desired characteristics in terms of particle size, shape, elemental composition, and chemical stability, making them invaluable for many applications [1–4]. Platinum (Pt) is one of the most important metals and as nanoparticles they have been extensively used in many applications such as catalysts in fuel cells [5–8] and in the exhaust systems of cars [8,9], gas sensors [8,10], glucose sensors [11], and cancer therapy [12]. In fuel cells, Pt nanoparticles have been shown to be an effective single component heterogeneous catalyst for the production of hydrogen from water in proton-exchange membrane fuel cells [5] as well as for dehydrogenation of methanol in direct methanol fuel cells [6]. Both bimetallic Pt-based alloy nanoparticles and core-shell nanoparticles have been studied for more effective heterogeneous catalysts in the development of efficient energy technology [7]. In catalytic converters, Pt nanoparticles are used to combine carbon monoxide and unburnt fuel from a car’s exhaust with oxygen from the air, forming carbon dioxide and water [9]. The catalytic efficiency and selectivity increases with decreasing particle size of nanometer dimensions [13]. In gas sensors, a component based on a platinum-tin oxide-silicon nitride-silicon dioxide-silicon metal-insulator-semiconductor capacitor has been developed, which is capable of detecting oxygen and carbon monoxide gases [10]. Fine Pt nanoparticles can be deposited on functionalized multi-walled carbon nanotubes (MWNTs) and these enzymatic Pt/MWNTs have been used as a glucose sensor [11]. Pt also has slight toxic effects on human cells and is therefore used for commercial cancer therapy [12]. With the help of core-shell nanoparticles, a selective effect on tumor cells could be achieved.

The properties of Pt nanoparticles depend strongly on the particle size, shape, elemental composition, and structure which can be controlled in the fabrication process. There are numerous reports concerning the synthesis of size-controlled Pt nanoparticles.The common approaches for the fabrication of colloidal Pt nanoparticles have been the chemical reduction method [14–16], polyol method [17], reverse micelles [18], electrochemical method [19], two-phase liquid-liquid route [20], microemulsions [21], green synthesis [22], photoreduction process [23] and gamma irradiation [24]. Among the various conventional methods, the gamma irradiation method offers several advantages. It is a simple procedure that produces fully reduced and highly pure metal nanoparticles, which are free from by-products or reducing agents [24–26]. The radiolytic method has been used for the preparation of bimetallic nanoparticles of Ag-Pt [27] and Al-Ni [28]. Interestingly to note is that the radiolytic synthesis can also be used for the production of a core-shell system as in the case for Pt nanoparticles coated with functional and soluble polymeric shells [29].

Metallic nanoparticles display strong optical absorption phenomena presumably owing to coherent oscillations of conduction band electrons in resonance with the electromagnetic UV–visible light, commonly known as the Surface Plasmon Resonance (SPR) [30–32]. The action of these conduction electrons regulates the physical and chemical properties of metal nanoparticles and an understanding of the optical absorption phenomena could provide an invaluable insight into the functions of metal nanoparticles. For example, 10-nm diameter silver and gold nanoparticles exhibited steady absorbance peaks centered at about 415 and 520 nm respectively. However, the optical absorption spectra of some noble metals such as Pt nanoparticles have not been consistently observed and they remain ambiguous. Previously, it was reported that Pt nanoparticles, in aqueous solution, had absorption spectra that stretched across the whole of the UV–visible region [33]. Also, it was reported that the chemically or electrochemically synthesized Pt nanoparticles exhibited a single unsteady absorption peak in the UV spectrum region [19,34]. The case of Pt nanoparticles exhibiting two unsteady absorption peaks at about 220 and 260 nm in the UV spectrum region has also been reported [35–44]. However, upon increasing the induction time, the absorbance of both peaks disappeared completely and a new absorbance peak emerged in the visible spectrum region at about 500 nm [42–44].

In this paper, we report the gamma radiolytic synthesis of colloidal Pt nanoparticles from platinum tetraammine complex ions (Pt(NH_3_)_4_Cl_2_) in an aqueous solution of polyvinyl pyrrolidone (PVP), which produced steady spectra with two absorption peaks. We also calculated the absorption spectra of an isolated Pt nanoparticle by quantum mechanical treatment and the results suggest that the steady absorption peaks conceivably originated from the intra-band transitions of conduction band electrons of Pt nanoparticles. We investigated the influence of the dose on the particle size as well as on the optical properties associated with the origins of conduction electrons of Pt nanoparticles.

## 2. Results and Discussion

### 2.1. Mechanism of Pt Nanoparticles Formation

**Figure 1:**
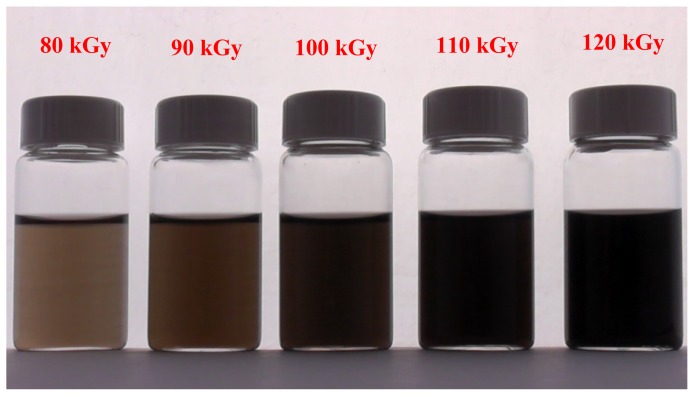
Photographs of colloidal Pt nanoparticles synthesized by the gamma radiolytic reduction method at doses of 80 to 120 kGy showing the colour of the colloidal solutions gradually turning from brown to dark brown, indicating evidence for the formation of Pt nanoparticles.

The colour of the colloidal solution changed from colorless before irradiation to dark brown after irradiation, indicating the formation of Pt nanoparticles. At higher doses the colour became even darker as shown in Figure 1.

**Figure 2:**
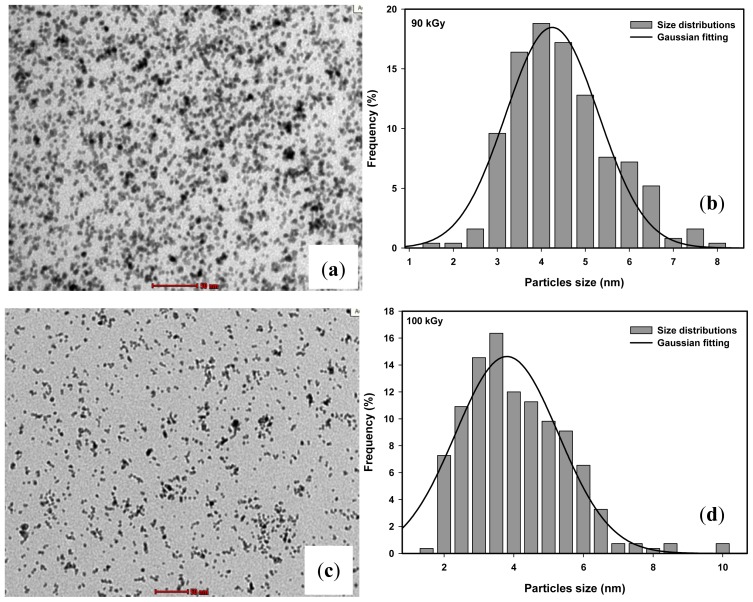
TEM images of colloidal Pt nanoparticles synthesized by the radiolytic method at doses of (**a**) 90 and (**c**) 100 kGy and histogram and Gaussian fitting of (**b**) 90 and (**d**) 100 kGy.

The TEM images of the particle distribution of Pt nanoparticles synthesized by the gamma radiolytic reduction method are shown in Figure 2a, and 2c for doses of 90 and 100 kGy respectively. The TEM images indicate well-dispersed particles, which are spherical in the size range of 2–10 nm. The average particle size of Pt nanoparticles synthesized at 90 and 100 kGy as determined from the Gaussian fitting of the size histogram, shown in Figure 2b,d, to be 4.2 and 3.8 nm respectively.

**Figure 3:**
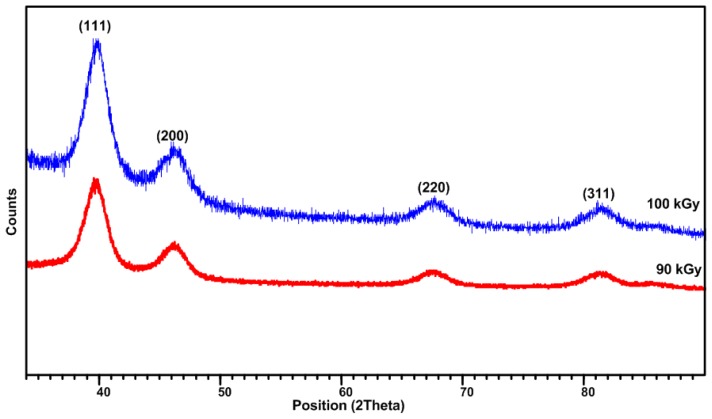
XRD patterns of colloidal Pt nanoparticles synthesized by the radiolytic method at doses of 90 and 100 kGy showing 2θ values of 39.8, 46.2, 47.5, 67.5, and 81.4 which match perfectly with the (111), (200), (220), and (311) crystalline planes respectively for the face centered cubic structure.

The formation of Pt nanoparticles can be observed by the colour change of the solution from colorless to dark brown (Figure 1) and from TEM images, which were confirmed by XRD studies. Figure 3 shows the XRD patterns of Colloidal Pt nanoparticles synthesized by the radiolytic reduction method at doses of 90 and 100 kGy. The XRD peaks were observed at 2θ values of 39.8, 46.2, 47.5, 67.5, and 81.4 which match perfectly with the (111), (200), (220), and (311) crystalline planes respectively for the face centered cubic structure of Pt (ICDD PDF 70-2431) with crystal lattice parameter of 3.924 A^o^ with lattice cell volume of 60.42 A^o3^. The mean crystalline size of Pt nanoparticles may be estimated from the width of the XRD peak using Scherrer’s equation [45,46] given by:

(1) $$D=\frac{k\lambda }{\beta \;\text{cos}\;\theta }$$

where *D* is the average crystallite size, *k* is the particle shape factor that varies with the method of taking the width and shape of crystallite (*k* = 0.89) [45], λ is the X-ray wavelength used (0.1542 nm), β is the angular line width of half-maximum intensity, and θ is the Bragg’s angle in degrees. The average crystalline sizes of the Pt nanoparticles were calculated using (111) reflection of the XRD patterns for Pt nanoparticles synthesized at 90 and 100 kGy and found to be 5.3 and 4.4 nm respectively, which were slightly higher than that obtained from TEM images [47].

In the radiation synthesis method, 1.25-MeV gamma rays interact with matter by photoelectric absorption, Compton scattering, and pair production. These results on the formation of secondary electrons, which in aqueous solution, induce among other types, hydrated electrons (e^−^_aq_), hydroxyl radicals (OH^•^), and hydrogen radicals (H^•^) by radiolysis of water according to Equation 2. The formation of Pt nanoparticles can be described by the following reactions. In solution, platinum tetraammine chloride dissociates into positive ions of [Pt(NH_3_)_4_]^2+^ and negative ions of 2Cl^−^, Equation 3. The hydrated electrons e^−^_aq_ reduce [Pt(NH_3_)_4_]^2+^ ions to zero valent Pt atoms (Pt^0^) by the first nucleation process, Equation 4.

(2) $${\text{H}}_{2}\text{O}\overset{\gamma -\text{rays}}{\to }{\text{e}}_{\text{aq}}^{-},{\text{H}}^{+},{\text{H}}_{3}{\text{O}}^{-},{\text{H}}^{\cdot },{\text{OH}}^{\cdot },{\text{H}}_{2},{\text{H}}_{2}{\text{O}}_{2}\;(\text{radiolysis of water})$$

(3) $$\text{Pt}{\left({\text{NH}}_{3}\right)}_{4}{\text{Cl}}_{2}\to {\left[\text{Pt}{\left({\text{NH}}_{3}\right)}_{4}\right]}^{2+}+2{\text{Cl}}^{-}\;(\text{ion dissociation})$$

(4) $${\left[\text{Pt}{\left({\text{NH}}_{3}\right)}_{4}\right]}^{2+}+2{\text{e}}_{\text{aq}}^{-}\to {\text{Pt}}^{0}+4{\text{NH}}_{3}\;(\text{reduction and nucleation})$$

Hydroxyl and hydrogen radicals (OH^•^ and H^•^), induced in the radiolysis of water, are also strong reducing agents in aqueous colloidal solution. To prevent this, isopropanol (IPA) was added into the precursor solutions. IPA scavenged OH^•^ and H^•^ radicals and at the same time was changed into IPA radicals, Equations 5 and 6, which eventually reduce [Pt(NH_3_)_4_]^2+^ ions into Pt^0^ as shown in Equation 7.

(5) $${\text{OH}}^{\cdot }+{\text{CH}}_{3}-\text{CH}(\text{OH})-{\text{CH}}_{3}\to {\text{CH}}_{3}-{\text{C}}^{\cdot }(\text{O})-{\text{CH}}_{3}+{\text{H}}_{2}\text{O }(\text{radical association})$$

(6) $${\text{H}}^{\cdot }+{\text{CH}}_{3}-\text{CH}(\text{OH})-{\text{CH}}_{3}\to {\text{CH}}_{3}-{\text{C}}^{\cdot }(\text{O})-{\text{CH}}_{3}+{\text{H}}_{2}\;(\text{radical association})$$

(7) $${\left[\text{Pt}{\left({\text{NH}}_{3}\right)}_{4}\right]}^{2+}+2[{\text{CH}}_{3}-{\text{C}}^{\cdot }(\text{OH})]\to 2[{\text{CH}}_{3}-\text{CO}-{\text{CH}}_{3}]+{\text{Pt}}^{0}+4{\text{NH}}_{3}+2{\text{H}}^{+}\;(\text{reduction and nucleation})$$

Since no reducing agent was employed, the process of reduction of Pt^0^ atoms by hydrated electrons or IPA radicals remains as long as the samples are irradiated with gamma rays. Many Pt^0^ atoms can agglomerate to form Pt^0^_2_ or Pt^0^_m+1_ nanoparticles, as shown in Equations 8 and 9 respectively. The agglomerated particle, Pt^0^_m+1_ can also combine with Pt(NH_3_)_4_]^2+^ ions, to form [Pt_m+2_(NH_3_)_4_]^2+^, Equation 10. Following the reduction process the ions change into larger Pt^0^_m+2_ nanoparticles, Equation 11. Large Pt^0^_m+2_ nanoparticles can agglomerate further with other Pt^0^ atoms to form even larger Pt^0^_m+n_ nanoparticles.

(8) $${\text{Pt}}^{0}+{\text{Pt}}^{0}\to {{\text{Pt}}^{0}}_{2}\;(\text{agglomeration})$$

(9) $${{\text{Pt}}^{0}}_{\text{m}}+{\text{Pt}}^{0}\to {{\text{Pt}}^{0}}_{\text{m}+1}\;(\text{agglomeration})$$

(10) $${{\text{Pt}}^{0}}_{\text{m}+1}+{\left[\text{Pt}{\left({\text{NH}}_{3}\right)}_{4}\right]}^{2+}\to {\left[{\text{Pt}}_{\text{m}+2}{\left({\text{NH}}_{3}\right)}_{4}\right]}^{2+}\;(\text{ion association})$$

(11) $${\left[{\text{Pt}}_{\text{m}+2}{\left({\text{NH}}_{3}\right)}_{4}\right]}^{2+}+2{\text{e}}_{\text{aq}}^{-}\to {{\text{Pt}}^{0}}_{\text{m}+2}+4{\text{NH}}_{3}\;(\text{reduction and agglomeration})$$

**Figure 4:**
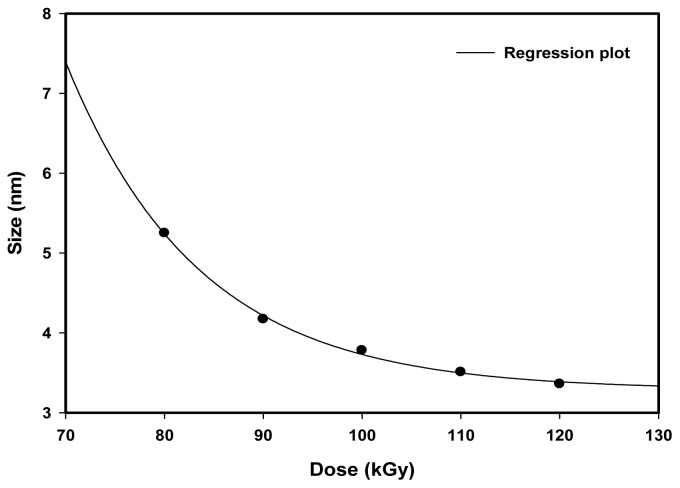
Average particle size of Pt nanoparticles was decreasing exponentially with increasing dose.

Figure 4 shows that the particle size decreased exponentially with the increase of absorbed dose from 80 to 120 kGy. The average particle size of each dose was determined at the peak of Gaussian fitting of the particle size histogram obtained from the TEM image using computer software. At low doses, the nucleation concentration is considerably lower than the concentration of unreduced [Pt(NH_3_)_4_]^2+^ ions. Thus, more Pt^0^ nanoparticles could be ionized by [Pt(NH_3_)_4_]^2+^ ions to form larger Pt^0^ nanoparticles following reduction and agglomeration. On the other hand at higher doses, most of the [Pt(NH_3_)_4_]^2+^ ions were consumed during nucleation processes. Hence, the nucleation concentration is considerably higher than that of the unreduced [Pt(NH_3_)_4_]^2+^ ions. Since most of the Pt^0^ nanoparticles were not ionized by [Pt(NH_3_)_4_]^2+^ ions, the Pt^0^ nanoparticles were consequently smaller in size at higher doses.

### 2.2. Optical Properties

**Figure 5:**
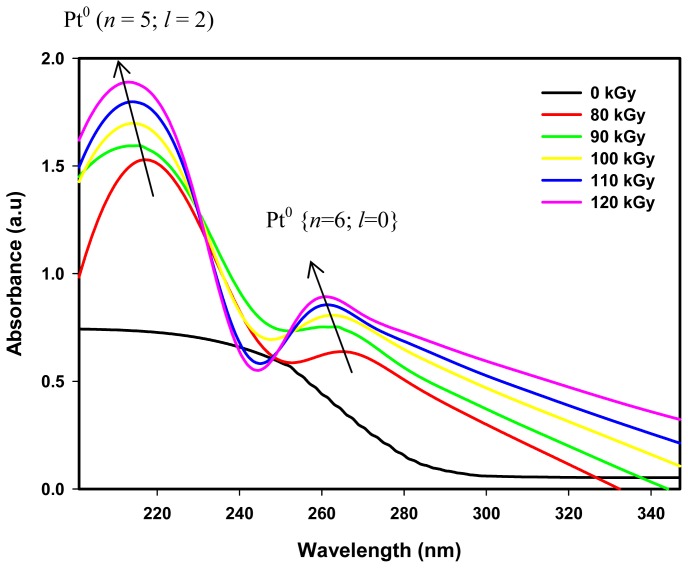
UV–visible absorption spectra of colloidal Pt nanoparticles synthesized by the gamma radiolytic method showing the evolution of two steady absorption peaks of 216 and 264 nm, in which the absorbance increased with increasing dose owing to the increased number of Pt nanoparticles with increasing dose. The absorption peaks blue shifted with increasing dose owing to a decrease in particle size.

Figure 5 reveals the evolution of two steady absorption maxima λ_max_ in the UV spectrum region centered at about 216 and 264 nm for colloidal solutions containing Pt nanoparticles synthesized by the radiolytic reduction method with doses of 80 to 120 kGy. The absorption maxima λ_max_ were however absent from the spectra of non-irradiated colloidal solutions, suggesting that Pt nanoparticles were not formed before irradiation. We also observed that both absorbance peaks of the Pt nanoparticles remain steady several weeks after irradiation indicating a complete reduction in the Pt nanoparticles formation. We also did not observe absorption peaks in the visible spectrum region for colloidal Pt nanoparticles synthesized by the radiolytic method.

In this experiment, Pt(NH_3_)_4_Cl_2_, PVP, IPA, tetrahydrofuran (THF), and deionized water were used. THF and deionized water were used as solvents for Pt(NH_3_)_4_Cl_2_ and PVP respectively. Irradiated polymers such as PVP and organic materials such as IPA and THF solvent can produce UV absorption peaks due to electronic transitions from HOMO to LUMO species. However, their absorption peaks were discounted by the spectrophotometer system leaving steady 216 and 264 nm absorption peaks, which we believed originated from Pt nanoparticles for two reasons: First, the absorbance of 216 and 264 nm absorption peaks increased with increasing dose due to the increase in the number of metal nanoparticles with increasing dose [26,28]. Secondly, the 216 and 264 nm absorption peaks blue shifted with increasing dose, indicating the peaks had shifted to lower wavelengths due to a decrease in the particle size (Figure 4). This behavior is well known for metal nanoparticles. Similar behavior associated with polymer or organic materials has not been reported.

In the radiolytic synthesis, the reduction process is accomplished after irradiation, which is unlike the chemical-based synthesis where the reduction process continues to happen at a slower rate or with longer induction times as long as the metal precursor or reducing agent has not been consumed completely. The disappearance of absorbance of the 220 and 260 nm peaks observed in chemically synthesized Pt nanoparticles owing to the reduction of PtCl_4_^2−^ and PtCl_6_^2−^ ions respectively [41,48] is no longer valid to describe the formation of steady absorption peaks of Pt nanoparticles synthesized by the radiolytic method. This is because in the radiolytic synthesis, no absorption peaks are observed before irradiation and the steady peaks of 216 and 264 nm only appear after Pt nanoparticles have been formed upon irradiation (Figure 5). There was a possibility that Pt(II) and Pt(IV) ion complex formation in colloidal solution increases with increasing dose. Details of the study of the possibility of the shifting of absorption peaks with increasing the dose could be explored using chemical instruments such as nuclear magnetic resonance and infra-red spectroscopy. However, we anticipated that the size of the ion complexes does not change with the dose to be able to give a blue shift of the absorption peaks (Figure 5). In our case, the absorption peaks blue shifted with increasing dose due to a decrease in the size of Pt nanoparticles with increasing dose. Moreover, the formation of Pt nanoparticles was established by TEM and XRD studies.

Hence, the absorption peaks of 216 and 264 nm possibly come from the conduction band of electrons that excite upon interaction with the electromagnetic field of UV–visible light. In our case, the absorption peaks of 216 and 264 nm conceivably originated from the intra-band transitions of the conduction band electrons of (*n* = 5, *l* = 2) and (*n* = 6, *l* = 0) energy states to higher energy states. To study this possibility, we calculated the absorption spectra of Pt nanoparticles of spherical diameter 5.3 and 3.8 nm, which were of similar dimensions to the average particle sizes of colloidal Pt nanoparticles produced by the radiolytic method at 80 and 100 kGy respectively.

As in semiconductor nanoparticles or quantum dots it is convenient to express the inter-band transitions of electrons from the valence band of the metal component to the conduction band of the non-metal component by quantum mechanical calculations [49–53]. Here we used a quantum mechanical treatment, as discussed in the theoretical section of Section 4, to establish the absorption peaks of 216 and 264 nm, which conceivably originated from the intra-band transitions of conduction band electrons of (*n* = 5; *l* = 2 or 5 d) and (*n* = 6; *l* = 0 or 6 s) energy states in transit to higher energy states. Details of the quantum mechanical treatment of the present theory of metal nanoparticles were based on our earlier published works [54,55] on Ag and Au nanoparticles, which possess the lowest conduction energy states of (*n* = 5; *l* = 0) and (*n* = 6; *l* = 0) respectively. Both nanoparticle systems require only one iteration process and produce a single absorption peak. However, for Pt nanoparticles which possesses the two lowest conduction energy states of (*n* = 5; *l* = 2) and (*n* = 6; *l* = 0), the iteration was performed twice, once for (*n* = 5; *l* = 2) state transitions and another for (*n* = 6; *l* = 0) state transitions. Other parameters required for the calculation of absorption spectra of Pt nanoparticles are the particle size, Pt atomic number (*Z* = 78), Pt Fermi energy (*E*_0_ = 9.74 eV) and lattice constant (0.393).

**Figure 6:**
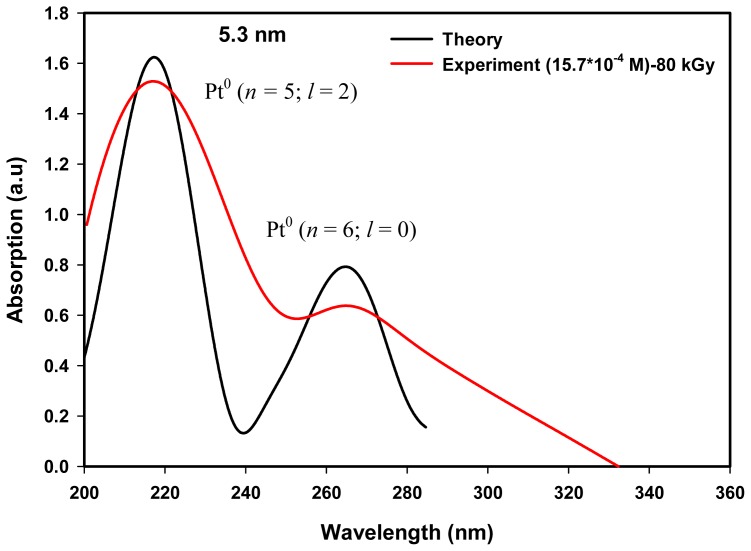
Absorption spectra with absorption maxima of 217.1 and 265.1 nm calculated for an isolated Pt nanoparticle of diameter 5.3 nm. The absorption peaks of 217.1 and 265.1 nm are attributed to the intra-band electronic transitions from conduction band energy states of (*n* = 5; *l* = 2) and (*n* = 6; *l* = 0) to higher energy states of (*n* ≥ 6; Δ*l* = 0, ±1; Δ*s* = 0, ±1) and (*n* ≥ 7; Δ*l* = 0, ±1; Δ*s* = 0, ±1) respectively, allowed by the principle of quantum numbers. Also shown is the measured UV absorption spectra with absorption maxima of 216.6 and 264.6nm of colloidal Pt nanoparticles synthesized at 80 kGy which produced Pt nanoparticles of average size of 5.3 nm (Table 1).

Figure 6 reveals the calculated absorption spectra showing the absorption maxima of 217.1 and 265.1 nm for an isolated Pt nanoparticle of diameter 5.3 nm. The first peak of 217.1 nm is attributed to the intra-band electronic transitions from the lowest conduction energy state of (*n* = 5; *l* = 2) to the higher energy states of (*n* ≥ 6; Δ*l* = 0, ±1; Δ*s* = 0, ±1). The second peak of 265.1 nm is attributed to the intra-band electronic transitions from the energy state of (*n* = 6; *l* = 0) to the higher energy states of (*n* ≥ 7; Δ*l* = 0, ±1; Δ*s* = 0, ±1). All possible intra-band electronic transitions allowed by the principle of quantum numbers would produce about the same absorption peak for a given spherical diameter since the energy states near the Fermi level are very close to or overlapping each other [54,55]. The present calculated absorption spectra shown here were represented by one of the electronic transitions. Also shown are the measured UV–visible spectra with the absorption maxima of 216.6 and 264.6 nm for colloidal Pt nanoparticles synthesized by the radiolytic method at 80 kGy, which produced Pt nanoparticles of an average particle size of 5.3 nm (Table 1). It is obvious that the experimental and theoretical absorption spectra (in arbitrary units) are not comparable to each other in terms of the height of the maximum and the width of the peak because the experimental data were obtained from many Pt nanoparticles with an average size of 5.3 nm, while the calculated data were based on a single Pt nanoparticle of diameter 5.3 nm. The most important information here is that the absorption peaks simulated for a spherical diameter of 5.3 nm were coincidently in good agreement with the absorption peaks measured for Pt nanoparticles synthesized at 80 kGy. The coincident agreement between the theoretical and measured absorption peaks indicates that the measured absorption peaks conceivably originated from the same intra-band transitions of conduction electrons from energy states of (*n* = 5; *l* = 2) and (*n* = 6; *l* = 0) to higher energy states of the conduction bands of Pt nanoparticles with particle size of 5.3 nm.

**Figure 7:**
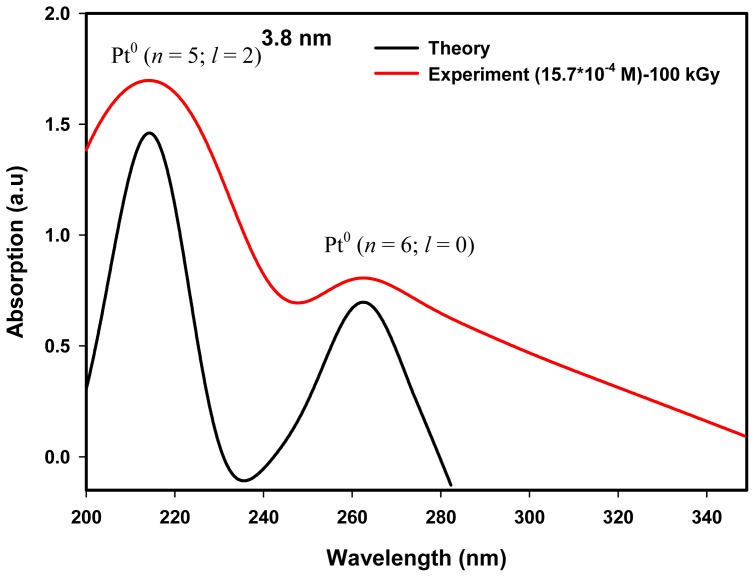
Absorption spectra with absorption maxima of 214.8 and 263.0 nm calculated for an isolated Pt nanoparticle of diameter 3.8 nm. The absorption peaks of 214.8 and 263.0 nm are attributed to the intra-band electronic transitions from conduction band energy states of (*n* = 5; *l* = 2) and (*n* = 6; *l* = 0) to higher energy states of (*n* ≥ 6; Δ*l* = 0, ±1; Δ*s* = 0, ±1) and (*n* ≥ 7; Δ*l* = 0, ±1; Δ*s* = 0, ±1) respectively, allowed by the principle of quantum numbers. Also shown is the measured UV absorption spectra with absorption maxima of 214.8 and 262.3 nm of colloidal Pt nanoparticles synthesized at 100 kGy, which produced Pt nanoparticles of average size of 3.8 nm (Table 1).

Figure 7 shows the calculated absorption spectra showing the absorption maxima of 214.8 and 263.0 nm of Pt nanoparticles of diameter 3.8 nm. Also shown are the measured UV–visible spectra with absorption maxima of 214.8 and 262.3 nm for colloidal Pt nanoparticles synthesized at 100 kGy which produced Pt nanoparticles of an average particle size of 3.8 nm (Table 1). The absorption peak of 214.8 nm is attributed to the intra-band electronic transitions from conduction band of (*n* = 5; *l* = 2) energy state to higher energy states of (*n* ≥ 6; Δ*l* = 0, ±1; Δ*s* = 0, ±1), and the absorption peak of 263.0 nm is attributed to the intra-band electronic transitions from conduction band (*n* = 6; *l* = 0) energy states to higher energy states of (*n* ≥ 7; Δ*l* = 0, ±1; Δ*s* = 0, ±1), allowed by the principle of quantum numbers. The absorption maxima of 214.8 and 262.3 nm of the synthesized Pt nanoparticles possibly come from the same intra-band electronic transitions from energy states of (*n* = 5; *l* = 2) and (*n* = 6; *l* = 0) to higher energy states of the conduction bands for Pt nanoparticle size of 3.8 nm. As can be seen from Figures 6 and 7 the height of the calculated peaks of 5.3-nm size are higher than those of the calculated peaks of 3.8-nm size simply because the number of atoms or conduction electrons of 5.3-nm particle size used for iteration is more than the number of conduction electrons of 3.8 nm particle size.

Table 1 shows the measured absorption peaks blue shifted from 216.6 to 212.7 nm for the first peak and from 264.6 to 260.9 nm for the second peak, when the dose increased from 80 to 120 kGy. Also illustrated are the calculated absorption peaks, which blue shifted from 217.1 to 212.3 nm for the first peak and from 265.1 to 261.7 nm for the second peak, when the particle size decreased from 5.3 to 3.4 nm. The calculated data are coincidently in agreement with experimental data. The reason for the blue shifts according to the present metal nanoparticles theory is that for the smaller particle size the number of atoms required to form a particle is few. Therefore, conduction electrons are less attracted to protons and thus increase the conduction band energy and decrease the absorption peaks.

Our results also shows that the absorbance at ~216 nm (the first peak) is always higher than the absorbance at ~264 nm (the second peak) as shown in Figure 5. Possibly this could be attributed to the concentration of conduction electrons of (*n* = 5; *l* = 2) energy state, which is nine times higher than that of (*n* = 6; *l* = 0) energy state (for each Pt atom there are nine electrons at (*n* = 5; *l* = 2) or 5 d orbit and one electron at (*n* = 6; *l* = 0) or 6 s orbit) as indicated in the theory (Figures 6 and 7). Therefore, the steady absorption peaks of 216 and 264 nm of Pt nanoparticles are conceivably derived from intra-band transitions of the conduction electrons originating at (*n* = 5; *l* = 2) and (*n* = 6; *l* = 0) energy states, respectively. The results suggest that according to the energy band theory of metals, Pt nanoparticles possess two conduction bands originating at (*n* = 5; *l* = 2) and (*n* = 6; *l* = 0) energy states.

**Figure 8:**
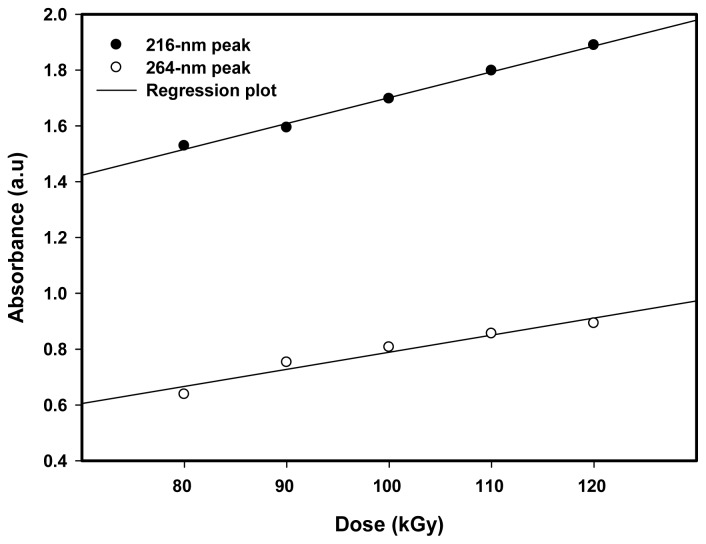
Absorption energy of Pt nanoparticles increased with increasing dose from 80 to 120 kGy owing to a reduction in particle size from 5.3 to 3.4 nm which corresponds to the change of the first absorption peak from 216.6 to 212.7nm and the second absorption peak from 264.6 to 260.9 nm (Table 1).

Figure 8 reveals the absorbance of absorption peaks of 216 and 264 nm, which increased linearly with increasing dose from 80 to 120 kGy. This means that the number of Pt nanoparticles increased with increasing dose [26,28]. As the dose increased from 80 to 120 kGy, higher nucleation processes take place and after agglomeration the formation of Pt nanoparticles increases. Moreover, as the dose increased from 80 to 120 kGy, the absorption peaks of 216 and 264 nm of the blue shifted towards lower wave lengths owing to a decrease in particle size from 5.3 to 3.4 nm (Table 1). This is because at higher doses the domination of the nucleation process over ion association allows smaller particle sizes to be formed with an increase in size distribution.

**Figure 9:**
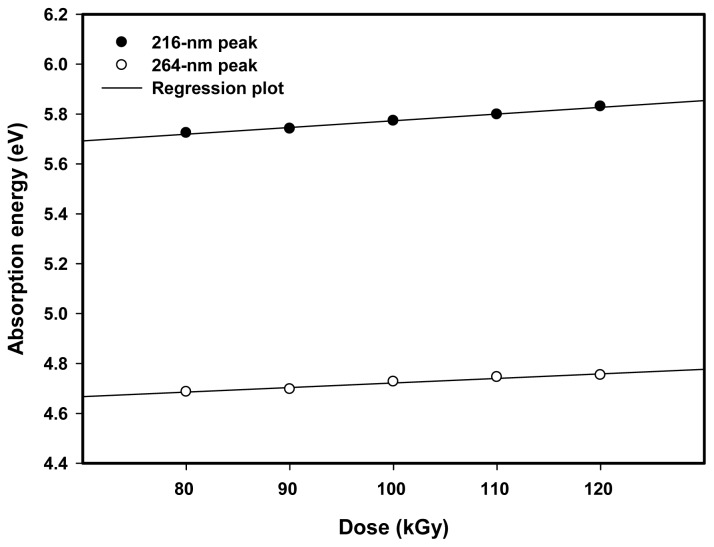
Absorption energy of 216 and 264-nm absorption peaks of Pt nanoparticles increased with increasing dose from 80 to 120 kGy owing to a reduction in particle size from 5.3 to 3.4 nm.

The absorption energy of Pt nanoparticles, *E* can be calculated according to *E* = *hc*/λ_max_, where *h* is Planck’s constant, *c* the speed of light, and λ_max_ the wavelength of the absorption maxima. The absorption energy or conduction band energy represents the amount of energy required to free conduction electrons from the attraction of Pt nanoparticles during excitation initiated by the electromagnetic UV–visible light. The 216 and 264-nm absorption peaks blue shifted towards lower wavelengths indicating the conduction band energy increased with increasing dose and decreased with increasing particle size (Table 1). Figure 9 shows the linear relationship between the absorption energy and dose for the 216 and 264 nm absorption peaks. The absorption energy increased from 5.72 to 5.83 eV for the first peak and from 4.69 to 4.75 for the second peak with increasing dose from 80 to 120 kGy due to a reduction in particle size from 5.3 to 3.4 nm (Table 1).

**Figure 10:**
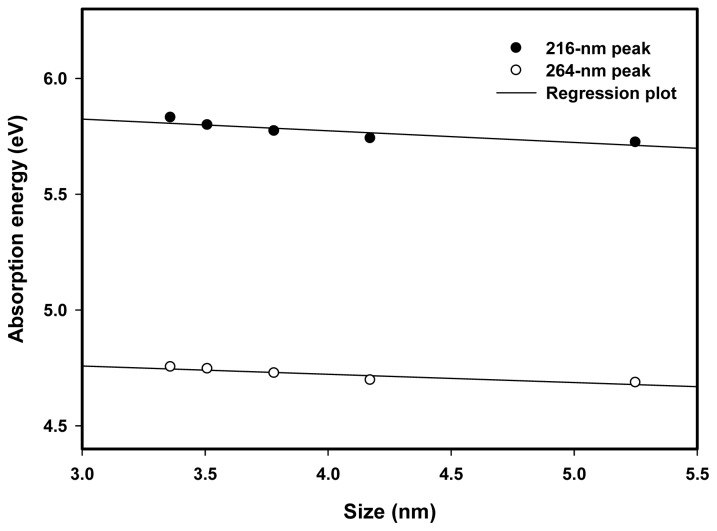
Absorption energy of Pt nanoparticles decreased from 5.83 to 5.72 eV for the first peak and from 4.75 to 4.69 for the second peak with increasing particle size from 3.4 to 5.3 nm which corresponds to the change of absorption peak from 212.7 to 216.6 nm for the first peak and from 260.9 to 264.6 nm for the second peak.

Figure 10 shows the relationship between the absorption energy or conduction band energy and particle size for the 216 and 264 nm absorption peaks. The conduction band energy decreased with increasing particle size due to the fact that for larger Pt nanoparticles the number of atoms is numerous, so that the conduction electrons are attracted to protons, which consequently reduces the conduction band energy of Pt nanoparticles. Our results suggest that Pt nanoparticles possess two conduction bands, which increased with decreasing particle size. These conduction band electrons strongly influence the optical and surface properties of Pt nanoparticles.

## 3. Experimental Section

Platinum tetraammine chloride hydrate, (Pt(NH_3_)_4_Cl_2_·H_2_O), polyvinyl pyrrolidone (PVP; MW = 29,000), isopropyl alcohol (IPA), and tetrahydrofuran (THF) were purchased from Sigma-Aldrich, St. Louis, MO, USA. All the chemical reagents were of research grade and used as received without further purification. Metal complex platinum tetraammine chloride was used as a metal precursor, PVP was used as a capping agent to reduce the agglomeration of Pt nanoparticles, IPA as radical scavengers of hydrogen and hydroxyl radicals, and THF and deionized water as solvents. 0.56 g of Pt(NH_3_)_4_Cl_2_·H_2_O was dissolved in 50 mL THF and 3.0 g of PVP was dissolved in 150 mL deionized water. The two solutions were separately stirred vigorously prior to mixing and then homogenized in the presence of IPA by further vigorous stirring for 1 h. Nitrogen (99.5% pure) was bubbled through the homogeneous solution for 1 h before dividing into six glass-tubes of which each contained about 33 mL. Each sample containing Pt(NH_3_)_4_Cl_2_·H_2_O, PVP, IPA,THF, and water received different doses at 0 and from 80 to 120 kGy using a 1.25-MeV ^60^Coγ-rays source. At the same time we also irradiated a solution containing only PVP, IPA, THF, and water of the same concentration without Pt(NH_3_)_4_Cl_2_·H_2_O for similar doses from 80 to 120 kGy to be used as reference samples in the UV–visible absorption measurements. Only samples containing Pt(NH_3_)_4_Cl_2_·H_2_O, PVP, IPA,THF, and water changed color after gamma irradiation.

The average particle size and size distribution were determined from transmission electron microscopy (TEM) (Hitachi, H-7500, Tokyo, Japan) at an accelerating voltage of 100 kV. TEM samples were prepared by placing a drop of irradiated solution on a copper grid, followed by overnight drying under ambient conditions. The synthesized Pt nanoparticles were characterized by X-ray powder diffraction (XRD) at a scanning rate of 5°/min in 2θ range 30°–90° using a Philips X-ray diffractometer (N.V. Philips Analytical X-ray, Almelo, the Netherlands) with Cu Kα radiation (λ = 0.1542 nm). The optical absorption spectra were measured in the frequency range of 200 and 800 nm using a UV–Visible spectrophotometer (UV-1650PC Shimadzu, Kyoto, Japan) on the next day after irradiation, allowing the IPA radical association and nucleation processes to complete. In the actual UV–visible absorption measurements, the monochromatic beam was split into two equal intensity beams. One beam, the sample beam passed through a transparent cuvette containing the irradiated sample (Pt(NH_3_)_4_Cl_2_·H_2_O, PVP, IPA, THF, and water) and the other beam, the reference beam, passed through an identical cuvette containing irradiated reference sample (PVP, IPA, THF, and water). All samples were diluted at the same dilution for the purpose of UV–visible absorption measurements. For un-irradiated samples, an un-irradiated solution containing PVP, IPA, THF, and water was used as reference sample. Prior to actual sample measurements, both cuvettes were filled with the correct reference sample to define the base line of the spectrophotometer system.

## 4. Theoretical Section

Hohenberg-Kohn-Sham density functional theory (DFT) has been most widely used to study the electronic structure of many-electron systems such as nanostructures. The early foundations of DFT are due to the Hohenberg and Kohn theorem [56] and the Kohn- Sham equations [57], where the ground state electron density ρ(***r***) is the basic variable, from which all ground state properties can be derived. For optical absorption of metal nanoparticles, the ground-state energy functional *E*[ρ(***r***)] may be taken from the Thomas-Fermi-Dirac-Weizsacker atomic model [57–62], written as

(12) $$E[\rho (r)]=T_{TF}[\rho (r)]+T_{W}[\rho (r)]+\int \rho (r)V(r)dr+J[\rho (r)]-K_{TFD}[\rho (r)]$$

where:

$T[\rho (r)]=\frac{3}{10}\frac{\hbar }{m}{\left(2\pi^{2}\right)}^{2/3}\int \rho {\left(r\right)}^{5/3}dr$ is the kinetic energy of the Thomas-Fermi (TF) model for the homogenous free electron gas system (conduction electrons) at a given coordinate ***r*** expressed in its original formulation of a local density approximation and expressed as a function of the electron density ρ(***r***),

$T_{W}[\rho (r)]=\frac{\lambda }{8}\frac{\hbar^{2}}{m}\int \frac{|\nabla \rho (r){|}^{2}}{\rho (r)}dr$ is the von Weizsacker correction to the kinetic energy of the TF model by inclusion of exchange and correlation energy terms for the inhomogeneous free electron density as a gradient correction to the uniform gas system with λ, the Weizsacker’s correction parameter,

$V(r)=\frac{Ze^{2}}{r}$ is the energy potential of free electrons due to the electric field of the protons in the metal nanoparticles without any electric and magnetic potentials,

$J[\rho (r,r^{\prime })]=\frac{1}{2}\iint \frac{\rho (r)\rho (r^{\prime })}{\left|r-r^{\prime }\right|}dr\;dr^{\prime }$ is the classical Coulomb energy of electron-electron interaction in the conduction band, and

$K[\rho (r)]=\frac{3}{4}{\left(\frac{3}{\pi }\right)}^{1/3}e^{2}\int \rho {\left(r\right)}^{4/3}dr$ is the Thomas-Fermi-Dirac (TFD) model, which refers to the non-classical exchange correlation energy of a homogenous free electron gas system, defined as containing all remaining quantum effects not captured by *J* and *T*. The lowest energy state of metal nanoparticles is the global minimum value E_0_ at the exact ground state density of ρ_0_, given by:

(13) $$E_{0}=E[\rho_{0}]=\text{min}\{E[\rho (r)]:\rho >0,\int \rho (r)\;dr=N\}$$

where *E*_0_ is the lowest (ground) state energy, *E*[ρ_0_] is the minimized energy function and *N* is the number of electrons in the conduction band of metal nanoparticles, which depends on the particle size. The ground-state electron density must satisfy the variational principle, δ{*E*[ρ(*r*)]−μ[∫ρ(*r*)*dr* − *N* ]}= 0 with μ = *E*_0_ is the Lagrange multiplier or Fermi energy at absolute zero temperature. The Euler-Lagrangian equation *E*[ρ(***r***)] of conduction band electrons of metal nanoparticles in terms of the second order differential equation is shown in Equation 14:

(14) $$\frac{1}{2}\frac{\hbar }{m}{\left(2\pi^{2}\right)}^{2/3}\int \rho {\left(r\right)}^{2/3}dr+\frac{\lambda }{8}\left[\frac{|\nabla \rho (r){|}^{2}}{\rho^{2}(r)}-2\frac{\nabla^{2}\rho (r)}{\rho (r)}\right]+V(r)+e^{2}\int \frac{\rho (r)}{\left|r-r^{\prime }\right|}dr-{\left(\frac{3}{\pi }\right)}^{1/3}\int \rho {\left(r\right)}^{1/3}dr=\mu$$

where ρ(***r***) is the density of conduction electrons of the Pt nanoparticle, the *E*_0_ = 9.47 eV is the Fermi energy of platinum, *r* is the displacement of conduction electrons from the center of the nanosphere, which is dependent on the Bohr radius α_0_, the atomic number *Z*, the principle quantum number *n*, the angular quantum number *l*, and the spin quantum number, *s* of the energy state. We consider the Pt nanoparticle as an isolated solid sphere of diameter *d* consisting of *N* number of atoms, confined in the face-centered cubic (fcc) lattice structure with a lattice constant of 0.393. We also consider that the conduction electrons are not absolutely free but weakly attached to the atom at the lowest energy state of the conduction bands of the Pt nanoparticles. According to the band theory of metals, the outer electron orbitals overlap into a conduction band, which can be occupied by conduction electrons. When light strikes a Pt nanoparticle, the conduction band electrons of ground energy states of (*n* = 5; *l* = 2) and (*n* = 6; *l* = 0) transit to higher energy states of the conduction bands. We found that the conduction electron density ρ(***r***) is a function of atomic number *Z* and absorption σ(***r***). The transformation of density functional energy *E*[ρ] into absorption functional energy *E*[σ] can be made algebraically with relatively simple mathematics involving only integration and differentiation. For numerical calculation, the absorption and wavelength of the final Euler-Lagrangian equation *E*[σ] were discretized. The multivariate equation may be solved by a trapezoid integration method using the Newton iterative program.

## 5. Conclusions

We synthesized colloidal Pt nanoparticles via the radiolytic reduction method, which produced average particle sizes in the range of 3.4–5.3 nm while the size increased with increasing the dose from 80 to 120 kGy. The Pt nanoparticles exhibited UV absorption spectra with two steady absorption peaks at 216 and 264 nm. The absorbance of the absorption peaks increased with increasing dose due to the higher nucleation process, which after agglomeration increases the number of Pt nanoparticles. The absorption peaks of 216 and 264 nm were investigated and possibly originated from the intra-band transitions of conduction electrons of (*n* = 5, *l* = 2) and (*n* = 6, *l* = 0) energy states respectively to higher energy states as established by quantum mechanical calculations. The conduction band energy derived from the absorption peaks increased with increasing dose and decreased with increasing particle size.

## Figures and Tables

**Table 1:** Average particle size, absorption peak, and absorption energy of Pt nanoparticles synthesized by the radiolytic reduction method at doses of 80 to 120 kGy.

Dose (kGy)	Particle size (nm)	First peak λ_max_ (nm)	Absorption energy (eV)	Second peak λ_max_ (nm)	Absorption energy (eV)
80	5.3	216.6 (217.1 *)	5.72	264.6 (265.1 *)	4.69
90	4.2	216.0 (215.9 *)	5.74	264.0 (263.9 *)	4.70
100	3.8	214.8 (214.8 *)	5.77	262.3 (263.0 *)	4.73
110	3.5	213.8 (213.9 *)	5.80	261.3 (262.1 *)	4.74
120	3.4	212.7 (212.3 *)	5.83	260.9 (261.7 *)	4.75

* calculated results.
